# Discovery and Domestication of New Fruit Trees in the 21st Century

**DOI:** 10.3390/plants11162107

**Published:** 2022-08-12

**Authors:** Hongwen Huang

**Affiliations:** Lushan Botanical Garden, Chinese Academy of Sciences, Jiujiang 332900, China; huanghw@scbg.ac.cn

The exploration and use of wild plant resources goes back to our rooted history of human civilization over about 20,000 years ago, before Ancient Mesopotamia in the Valley of the Tigris and Euphrates where barley, lentil and wheat were first domesticated [1]. From collecting wild fruits and nuts to domesticating cultivated crops during the agricultural civilization, the successful domestication and cultivation of every crop changed our civilization. Indeed, the crop plants have changed our life rather than we have successfully domesticated crop plants. Plant resources are of fundamental importance for human survival and development. A gene can impact a country’s economy, and a species can change a country’s rise and fall in prosperity [2]. However, our understanding of exploring and utilizing plant resources is only the tip of the iceberg. Ninety percent of the world’s food production comes from a couple dozen plants [3].

## 1. Fruit Domestication

The domestication of fruit crops has always been a long-term endeavor with terrible uncertainty and tremendous difficulty. In the 20th century, only four major fruit crops have been successfully domesticated from the wild, i.e., blueberry (*Vaccinium* L.), kiwifruit (*Actinidia* L.), avocado (*Persea* Mill.) and macadamia nuts (*Macadamia* F. Muell.). However, plant domestication is part of our civilization and has been a sustained and endless process throughout human history. In the beginning of the 21st century, the domestication of fruit crops has been arisen in many efforts of a few wild plants, such as *Akebia trifoliata* subsp. *australis* (Diels) T. Shimizu, *Lonicera caerulea* L., *Kadsura coccinea* (Lem.) A. C. Smith) etc. in China; *Asimina triloba* (L.) Dunal, etc. in North America; *Aristotelia chilensis* (Molina) Stuntz, etc. in South America [3].

In the history of fruit crop domestication [4], whether it is an apple, plum and grape etc. domesticated thousand years ago, or kiwifruit and blueberry etc. domesticated in the last century, a sustained purpose and clear domestication strategy have always been key factors for any successful domesticated fruit plants. Even nowadays in the 21st century in the domestication of new crop plants from the wild, the key towards success of the domestication of a new crop plant should be a long-term vision and well-formulated strategy of domestication, including clear goals of genetic improvement, an appropriate breeding approach and new breeding technologies employed. For example, a pioneer domestication program of *Akebia*, native to China in the wild, a well formulated strategy was drafted and put forward since the beginning of this century [5,6], with a set of six well-projected goals for domesticating improvements, including the traits of fruit such as, crackingness, fruit size, fruit peel thickness, seed numbers, fruit edible ratio and fruit storage [7]. This domestication program also integrates traditional breeding with current new technology, including selecting superior genotypes from wild populations, intraspecific and interspecific hybridization, recurrent and pedigree selection, molecular marker assisted selections and ploidy manipulation to improve the breeding efficiency.

Domestication of a new fruit plant may not be as easy as many plant breeders or pomologists thought. Apparently, apart from a well-projected goal of a set of traits to be genetically improved, germplasm resources in the starting point plays a crucial role for the success of domesticating a new fruit plant [8]. Among the hundreds of wild fruit plants with domesticating potential, only a few have been evaluated and used for serious domestication processes, exemplified by a general lack of understanding and efforts in germplasm evaluation at the starting stage of domestication. In fruit domestication, the human pursuit for new tasting fruits are endless. Of course, nowadays, people are increasingly pursuing fruits with a good taste, nutrition and health care. The introduction and domestication of new fruits is more inclined towards the exploration and utilization of wild fruit tree resources with high nutritional and therapeutic value. New fruit discoveries from the wild have obvious advantages in nutrition, therapeutic value and novel taste. However, compared with commercial fruits, there are some characteristics such as poor palatability, small fruit and low yield must to be improved to meet the market acceptability. An appropriate breeding and selection goal is very important for the domestication of wild fruit trees. Firstly, a broad spectrum of wild germplasm resources is systematically investigated, collected and evaluated to lay a solid foundation for their continuous variety improvement. Second, market feedback is one of the required endeavors for fruit tree breeders. The timely understanding of the market recognition of varieties can avoid the inertia of “self-entertainment” of breeders in the breeding orchards. From the perspective of domestication and improvement methods of wild fruit plants, conventional breeding methods are still the main technique in the initial domestication stage of new fruits, especially natural variation selection, intra and interspecific hybridization, systematic breeding and so on. With the advancement of domestication process, modern biotechnology assisted breeding and genetic manipulation can be used to speed up the domestication and genetic improvement process [9].

## 2. Germplasm Collections

Germplasm collections primarily provide the raw material for programs of any plant breeding and genetic improvement. Hundreds of living germplasm collections of fruit tree crops across the world have played an incredible role in fruit tree breeding and enabled many newly improved cultivars available to the worldwide fruit industry. Germplasm repositories for fruit trees are uniquely constructed as clonal living collections preserved in orchards, vineyards and plantations or nurseries, etc. including diverse valuable resources such as current commercial cultivars, traditional cultivars, landraces, elite lines, and other breeding materials or wild relatives [10,11]. In addition to the preservation and maintenance of well-documented germplasm, fruit tree living collections are not only used by fruit breeders but other biologists who may have different interests and possibly different inquiries. While fruit breeders focus on commercial traits of immediate perceived value, other biologists may be more interested in investigating the properties and behaviors of the plant, domestication history, evolutionary phylogeny, especially at the genomic level.

One of the prioritized tasks in a germplasm repository of fruit tress is to characterize the genetic diversity and composition of accessions maintained in the repository to capture an as broad as possible genetic and morphological diversity, to facilitate the characterization of intra- and interspecific variation, to understand phylogenetic relationships among all resources including cultivars, varieties, subspecies and species, particularly those between wild relatives. The quantity and quality of data documentation are of crucial importance for germplasm repository management. Although data documentation changed over time with initial morphological evaluation, genomic data have been increasingly obtained from a wide range of fruit tree germplasm collections. Genotyping accessions have become routine protocol in fruit tree germplasm collections to verify pedigrees and track a trait of interest in breeding. Nevertheless, genetic information by using SSR, SNP and AmpSeq, etc. provides valuable a resource for breeding designs of fruit trees regardless of traditional breeding or molecular breeding programs, in particularly, parentage choice. The nature of fruit trees as mostly perennial plants also provides other biologists the ability to study and understand many aspects about basic plant biology, such as growth, development, reproduction, biotic and abiotic stress tolerance, as well as metabolites changes etc. due to germplasm repository of fruit trees maintained in the same location across multiple years, which offer important opportunities to study inter-annual variation under common-garden conditions. One approach, for example, is to study phenotypic plasticity in response to climatic changes and adaptive evolution of long-lived plants.

Ultimately, fruit tree living collections are of particular use for fruit tree breeding and genetic improvement for new cultivar development, for example, many new kiwifruit cultivars recently released to the worldwide kiwifruit industry rely heavily on the Chinese National Kiwifruit Germplasm Repository at the Wuhan Botanical Garden, Chinese Academy of Sciences. Recently, molecular approaches such as GWAS, QTLs and other marker-assisted selection have been increasingly used as tools for selecting potential parents from germplasm collections for use in many fruit tree breeding programs to accelerate the breeding process [12]. It is evident that fruit tree improvement could greatly be enhanced by standardized and statistically robust procedures for discovering quantitative trait loci (QTLs) in germplasm relevant to breeding programs and aided for validation of important breeding parents (IBPs) by estimating average allelic representation in wild relatives, which also demonstrated the importance of constructing a core germplasm set in fruit tree living collections [11].

Looking to the future, what new fruit crops could be domesticated in the new century? When economical and societal development are rapidly evolving, the elevation of living standards and awareness of health care have increasingly challenged fruit breeders for novel and high nutritional or functional fruits that are of benefit to human health. The new fruit types are becoming increasingly in high demand in the marketplace. No doubt fruit breeders will continue to seek new possibilities to domesticate new fruit crops. Apart from their ongoing improvement of current fruit crops, fruit germplasm curators put their efforts in the discovery of new potential fruit crop germplasm sources from collections or in the wild. Domesticating new plant crops is in fact never stalled, but an ever-continuous process in our mankind civilization and history. The incentive was originally driven by the endless pursuit for new taste of fresh fruits, but evolved to the preference of taste, nutrition and health care. Fundamentally, the domestication of new fruits relies on the discovery of the wild fruit plants with high nutritional and food therapeutic value.

## 3. Ex Situ Flora for the Sustainable Future

The planetary scale changes characterizing the Anthropocene have substantially transformed natural vegetation for millennia, most notably as a result of the Columbian exchange in the past 500 years [13,14], when increasing globalization and agricultural expansion has led to dramatic plant species extinction and an increasing homogenization of global biodiversity [15,16]. In this context, plant conservation in the Anthropocene is extremely challenging and despite strenuous efforts, the Aichi Biodiversity Targets have not been met [17]. Now, the emerging post-2020 global biodiversity framework (GBF) is under fire over the setting of hotly debated goals, while the deep philosophical connections between humanity and biodiversity inevitably need to be addressed. From the historical hunting and gathering for food and drugs from plants to today’s active efforts to cultivate rare and threatened plants. Germplasm, botanists, gardeners and living plant germplasm curators acting as anthropogenic agents of dispersal have generated extraordinarily diverse ex situ floras in marked contrast to the less than a quarter of the unused wild and mostly fragmented flora remained in situ around the globe.

Currently, there are ca. 30% of the world’s known plant species conserved in botanical gardens or other germplasm living collections around the world [18], highlighting the global socioeconomic importance of anthropogenic ex situ plants. As a key example, the ex situ Flora project in China, which was initiated in 2012 [19], was the first extensive inventory of ex situ Flora by any megadiverse country of the plants conserved in ex situ collections. The project has resulted in the 20 published volumes of the ex situ Flora of China and brought together a combined mega-database of the living plant inventory. Analyses of this database reveal that the ex situ Flora of China comprises of more than 28,000 species belonging to >3500 genera and >310 families, approximately equating to >7% of known plant species in the world. More than half of the plant species comprised in the ex situ Flora of China (c. 16,000, 57%) are native to China and together they account for 53% of China’s known native vascular plants (30,471) and about 4% of the world’s total vascular plant flora (387,193 species, http://wcvp.science.kew.org/, accessed on 8 May 2022). Among the total, there are ca. 4000 plants with known uses or economic value, by one measure, accounting for ca. 24% of the world’s known economically important plants [20]. In addition, there are more than 400 plant species verified as edible fruits, belonging to 58 families and 132 genera [http://doi.org/10.13430/j.cnki.jpgr.20210902001, accessed on 8 May 2022]. Phylogenetically, at the generic level, the ex situ Flora of China compared to the world’s flora and all vascular plant genera in China shows that the genera are most phylogenetically clustered, suggesting a general integrity of phylogenetic patterns. The non-native species conserved in Chinese botanical gardens comprise of about 43% (c. 12,000) of the total ex situ species conserved (28,000). The non-native genera (>1000 genera of the world total) fill in the phylogenetic gaps of missing native genera (c. 900 genera) although the ex situ flora is distorted toward the lineages of tropical genera per economically important exotic plants, including those from Africa (c. 2000 species), South America (c. 1800), North America (c. 1200), Asia-Tropical (c. 800), Asia-Temperate (c. 400), Europe (c. 350), Australasia (c. 200) and Pacific islands (c. 50).

The ex situ flora is an under-recognized but highly valuable natural and cultural heritages reflective of the diverse interactions between human and plants that are embedded in our anthropogenic history. Our heavily exploited and degrading planet is to a large extent a direct consequence of more than five centuries of great biological and cultural exchange that continues to the present day, which has resulted in patchy in situ flora but increased ex situ flora across both global and regional scales. Anthromes now cover more than half of Earth’s land [14], leaving about a quarter of wild flora still remaining in situ. Although it is a widely accepted that we need to conserve both our natural and cultural heritages, the consequences of Earth’s anthropogenic transformation with large artificially assembled ex situ flora but highly fragmented in situ flora are still not fully assessed and understood. A thorough inventory of the global ex situ flora is worth greater focus to address the global safeguarding of flows of plant resources from nature coupled with the science-based policies and strategies that are needed to forge sustainable futures.

While the Anthropocene is now driving plant species to extinction at rates of a hundred to a thousand times faster than normal [21], an increasingly large number of plant species in ex situ flora both globally and regionally remain unknown: how many species? exactly which are they? what is their status? are they potentially useful plant resources? what phylogenetic gaps remain and how might they be filled? A fifth of the twenty-first century is behind us already, and we are thirty years beyond Rio, yet natural vegetation, and the specialized plant habitats of which they are comprised continue to be lost or degraded. Our lists of threatened plants grow bigger, not smaller, and germplasm erosion worsens. The current contorted or imbalanced approaches to conservating plant diversity have been the subject of stern doubts and criticism globally, regionally and nationally. In situ conservation remains the optimal means of conserving ecosystems that approximate their pre-Anthropocene functioning, but at the same time, the contribution that ex situ conservation can make and the importance of ex situ living plant collections should not be overlooked, particular of fruit or many other crop ex situ living collections of germplasm for discovery and domestication of new crops in the 21st century.
